# Zone-specific damage of the olfactory epithelium under protein restriction

**DOI:** 10.1038/s41598-020-79249-3

**Published:** 2020-12-17

**Authors:** Ayinuer Tuerdi, Shu Kikuta, Makoto Kinoshita, Teru Kamogashira, Kenji Kondo, Tatsuya Yamasoba

**Affiliations:** 1 Department of Otolaryngology and Head and Neck Surgery, The Second Xiangya Hospital, Central South University, 139 Renmin Road, Changsha, 410011 Hunan China; 2 Department of Otolaryngology and Head and Neck Surgery, Faculty of Medicine, University of Tokyo, 7-3-1 Hongo, Bunkyo-ku, Tokyo, 113-0033 Japan

**Keywords:** Auditory system, Olfactory system, Sensory processing, Health care

## Abstract

Oxidative stress causes tissue damage, affecting age-related pathologies. Protein restriction (PR) provides a powerful intervention strategy for reducing oxidative stress, which may have a positive effect on individual organs. However, it is unknown whether PR intervention influences the olfactory system. Here, we investigated how 10 months of PR could affect the cell dynamics of the olfactory epithelium (OE) in mice. We found that PR reduced age-related loss of outer hair cells in the cochlea, providing preventive effects against age-related hearing loss. In contrast, PR resulted in reduced mature olfactory sensory neurons (OSNs), increased proliferative basal cells, and increased apoptotic OSNs in zone 1 (the only area containing neurons expressing NQO1 [quinone dehydrogenase 1]) of the OE in comparison with animals given a control diet. Substantial oxidative stress occurred in NQO1-positive cells and induced apoptotic OSNs in zone 1. These results indicate that in contrast to the positive effect on the auditory system, PR induces oxidative stress and structurally and functionally negative effects on OSNs in zone 1, which is probably involved in the bioactivation of NQO1.

## Introduction

Oxidative stress is a phenomenon caused by an imbalance in the production and detoxification rates of reactive oxygen species (ROS). High level of ROS cause extensive damage to proteins, DNA, and lipids, and thereby affect aging and age-related diseases including cancer^1^, neurodegeneration^2^, cardiovascular disease^3^, and diabetes^4^. Coordinated regulation against ROS production could be linked to the reduction of oxidative stress, leading to normal cellular function and organ homeostasis.

Nutritional interventions such as calorie restriction (CR) and protein restriction (PR), which are non-genetic approaches, are strongly associated with longevity and metabolic health^5–7^. Indeed, specific nutrients, rather than overall calories, mediate the positive effects of dietary interventions involving protein and specific amino acids. PR reduces insulin/insulin-like growth factor (IGF) signaling at systemic levels, mitochondrial ROS generation, oxidative damage to mitochondria, and nuclear DNA in mammalian cells, providing sustained beneficial effects on individual organs above those achievable through CR^8–13^. Despite rising evidence for the various beneficial effects on metabolic health that can be attained through PR intervention, it is unclear whether PR intervention provides a positive effect on all organs.

As a result of its anatomical location, the olfactory epithelium (OE) is continuously exposed to a variety of potentially harmful air pollutants and endogenous neurotoxic compounds derived from normal cellular respiration and various metabolic reactions, which can damage olfactory sensory neurons (OSNs) in the OE. When OSNs are damaged and lost, there is prompt and massive regeneration of new OSNs through the proliferation and differentiation of progenitor cells, and these new OSNs are subsequently incorporated into olfactory neural circuits^14^. At the cellular level, OSNs contain various types of antioxidants and chemo-protective enzymes, such as NQO1 (NADPH quinone oxido-reductase 1), glutathione peroxidase, and superoxide dismutase (SOD)^15–18^. These endogenous enzymes may work to detoxify harmful agents and contribute to the maintenance of olfactory function. However, changes in nutritional status may induce energy metabolism changes at systemic levels, which will inevitably induce changes in metabolic byproducts at the cellular level^19^. This situation could profoundly and dynamically affect the physiological properties of endogenous enzymes responsible for detoxification, as well as the cell dynamics, in the OE^19,20^.

In the current study, we explored the histological and functional effects of long-term PR on the cell dynamics of the OE. We first set out to confirm that PR intervention positively affected age-related cell dynamics in the cochlea. In striking contrast, we found that PR intervention induced a significant decrease in OSNs in the dorsomedial area of the OE, which corresponds to zone 1 determined by co-localization with NQO1 expression. Increases in the numbers of proliferative basal cells and apoptotic OSNs were observed in zone 1. Furthermore, substantial oxidative stress occurred in NQO1-positive cells and induced apoptotic OSNs. These results indicate that PR could induce negative structural and functional effects on the olfactory system, particularly in zone 1 of the OE, and that area-specific injury may be involved in the bioactivation of NQO1.

## Results

**Figure 1:**
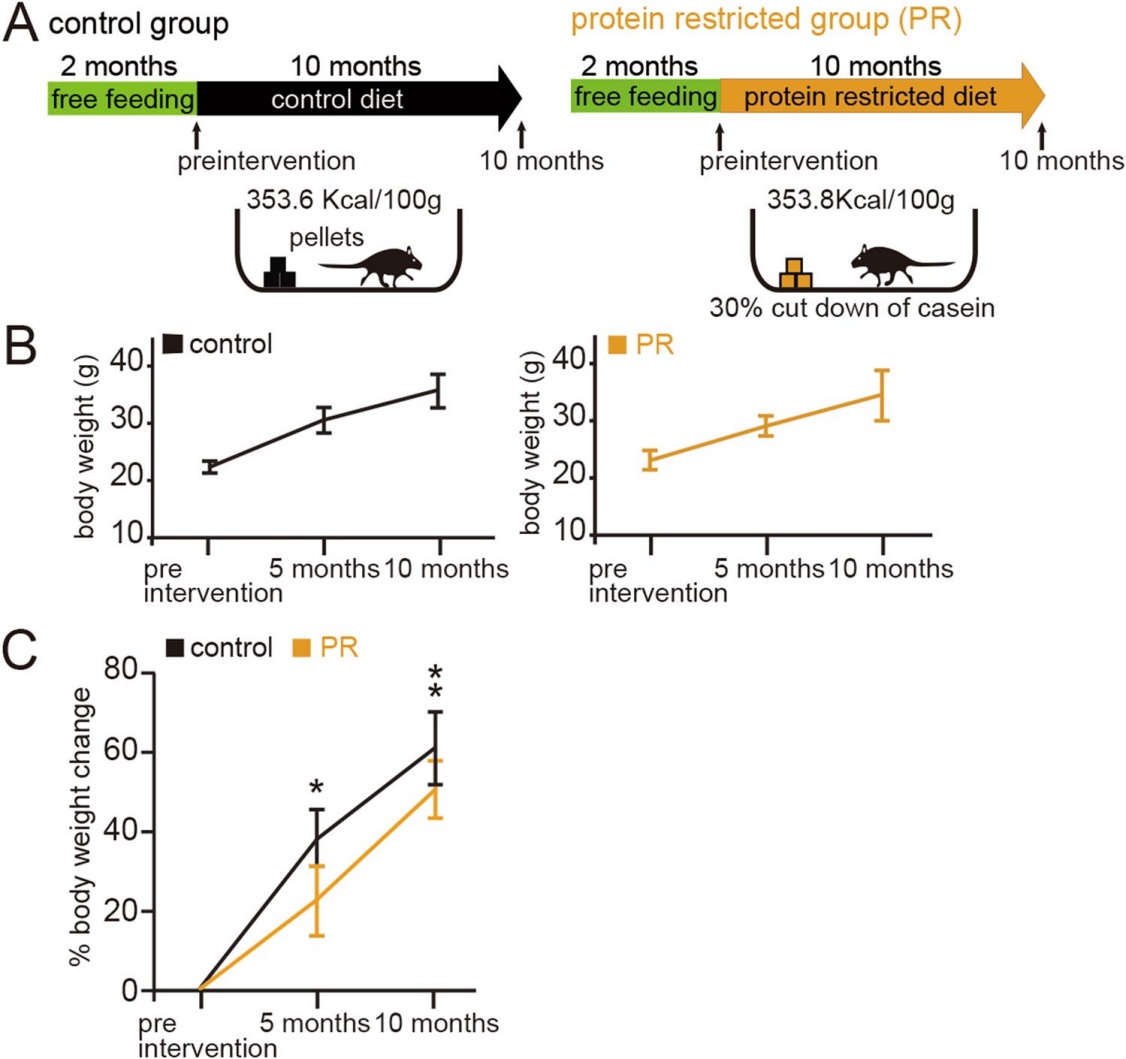
Effects of protein restriction on body weight. (**A**) Method of protein restriction (PR). Mice at 2 months old (preintervention) were divided into two groups: controls, fed a control diet for the following 10 months (353.6 kcal/100 g); and PR, fed a protein-restricted diet for the following 10 months (353.8 kcal/100 g). All mice were exposed to odors for c-fos experiments before fixation. (**B**) Body weights of control (left) and PR mice (right) at preintervention (2-month-old mice), 5 months (7-month-old mice), and 10 months (12-month-old mice) in each group (eight mice in each group). Data are shown as mean ± SD. (**C**) Percent body weight differences at preintervention, 5 months, and 10 months (eight mice in each group). At 5 and 10 months, a significant reduction in percent body weight change was observed in PR mice compared with control mice (*P < 0.05, **P < 0.01, Mann–Whitney test). Data are shown as mean ± SD.

The experimental regimens are shown in Fig. 1A. Briefly, 2-month-old mice (preintervention mice) were randomly divided into two groups: control and protein-restricted (PR) groups. Each group of mice was subjected to pellets containing a very similar energy content for the next 10 months, with the amount of pellets provided (approximately 4 g per day) being calculated on the basis of the daily food intake data. The PR mice were subjected to a protein-restricted diet for 10 months, in which the casein content was reduced by 30%, while the calorie intake was maintained (Table 1).

**Table 1:** Nutrient composition of the diet fed to the protein-restricted mice.

Nutrient composition	Control	PR
Casein	14.0%	9.8%
β-Cornstarch	46.6%	46.4%
α-Cornstarch	15.5%	19.7%
Sucrose	10.0%	10.0%
Soybean oil	4.0%	4.0%
Cellulose powder	5.0%	5.0%
AIN-93G mineral mixture	3.5%	3.5%
AIN-93 vitamin mixture	1.0%	1.0%
Cholinebitartrate+	0.3%	0.3%
Tertiary butyl hydroquinone	0.1%	0.1%
Total	100.0%	100.0%
Total calorie (kcal/100 g)	353.6	353.8

Control mice were fed with a diet of 353.6 kcal/100 g. Protein-restricted (PR) mice were fed with a diet of 353.8 kcal/100 g containing a 30% reduction in casein from AIN-93M, which was replaced with a cornstarch supplement.

The body weights of the control and PR mice gradually increased during the 10-month period (Fig. 1B). The percent change in body weight in the PR mice was significantly lower than in the age-matched control mice (5 months: P < 0.05, 10 months: P < 0.01; eight mice per group, Mann–Whitney test, Fig. 1C). These results indicate that PR reduced body weight gain compared with a control diet and could potentially influence metabolic energy systems.

### PR delays the loss of OHCs associated with age-related degeneration of the cochlear

**Figure 2:**
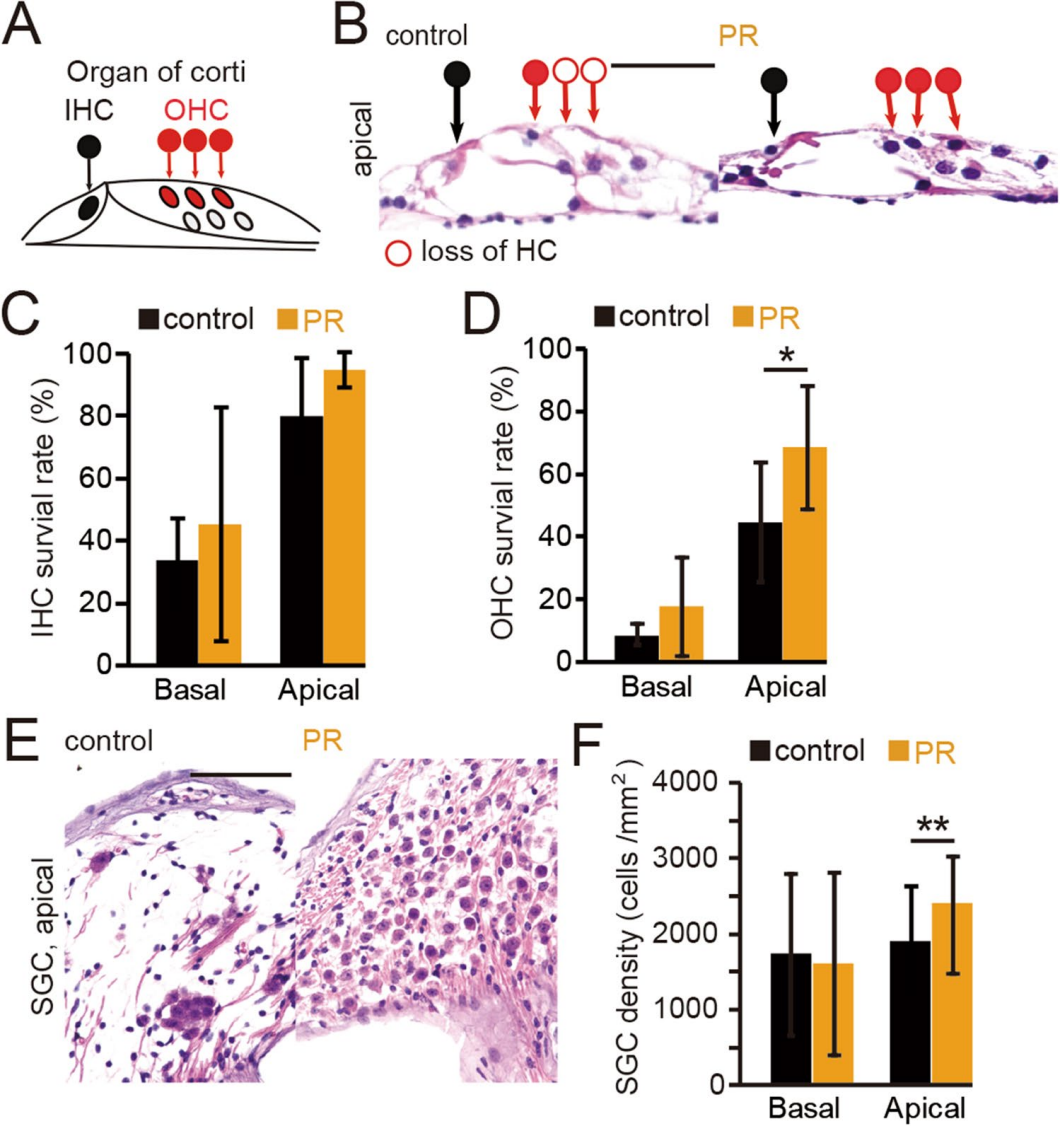
Effects of 10 months of PR on age-related degenerative changes in the inner ear. (**A**) Schematic diagram of the organ of Corti. The closed black circle indicates a surviving inner hair cell (IHC), and the closed red circle indicates a surviving outer hair cell (OHC). (**B**) Hematoxylin and eosin-stained images at the apical turn of the organ of Corti in control and PR mice. The open circle represents the loss of hair cells. Scale bar 50 μm. (**C**) Survival rates of IHCs in control and PR mice. At basal and apical turns, no significant differences in IHCs were found between PR and control mice (Mann–Whitney test). (**D**) Survival rates of OHCs in control and PR mice. The OHC survival rate in the PR mice was significantly higher than that in the controls only at the apical turn (*P < 0.05, Mann–Whitney test). (**E**) Representative images of spiral ganglion cells (SGCs) at the apical turn in control and PR mice. Scale 50 μm. (**F**) Summary of the SGC density at the basal and apical turns. Significantly higher averaged SGC density in the PR mice compared with the controls was observed only at the apical turn (**P < 0.01, Mann–Whitney test).

The auditory function of mice declines gradually in later life^21,22^. The histopathology of this decline consists of the loss of IHCs, OHC, and SGCs in the basal turn, with a progressive loss of OHCs and SGCs from the base towards the apical portion of the cochlea as animals age^21^. We therefore examined whether PR in the present experimental setting could delay age-related cochlear degeneration. We observed histological changes in IHCs, OHCs, and SGNs in both control and PR mice (Fig. 2A,B). In the analysis of IHCs and OHCs, we calculated the survival rates of HCs, i.e., the percentage of surviving HCs in the organ of Corti (see “Materials and methods”)^23^. In both control and PR mice, age-related degeneration of HCs was observed specifically in the basal turn compared with the apical turn (Fig. 2C,D). Such age-related degeneration of HCs was ameliorated in the PR mice compared with the controls, and this preventive effect was statistically significant in the OHCs of the apical portion (IHCs [basal turn]: P = 0.5; [apical turn]: P = 0.07; OHCs [basal turn]: P = 0.27; [apical turn]: P < 0.05; four mice per group, Mann–Whitney test, Fig. 2C,D). These results indicate that PR could prevent degenerative changes to the HCs, most predominantly in the OHCs within the apical turn. Degenerative changes of the SGCs were also ameliorated by PR in the apical turn (Fig. 2E,F), consistent with the histological changes observed in the HCs (SGCs [basal turn]: P = 0.36; [apical turn]: P < 0.01; four mice per group, Mann–Whitney test, Fig. 2F). These results showing that PR ameliorated age-related degeneration of OHCs and SGNs in the apical regions of the cochlea indicate that the PR used in the current study had a protective effect against age-related degeneration in the auditory system.

### Zone 1 of the OE was histologically and functionally injured under PR intervention

**Figure 3:**
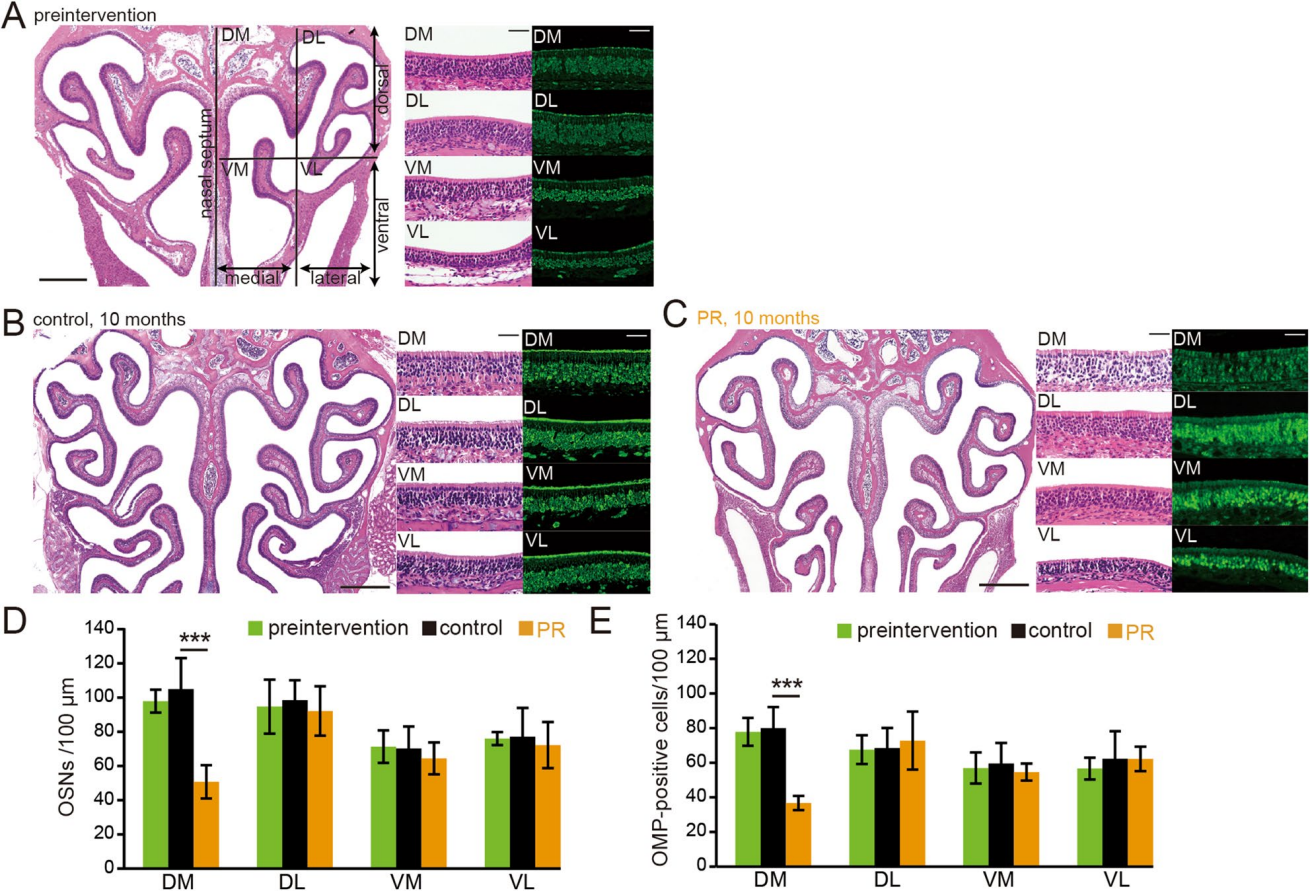
PR induces OE injury in the dorsomedial area of the OE. (**A**–**C**) Photomicrographs of representative coronal sections of the olfactory epithelium (OE) in preintervention mice (**A**), control mice (**B**), and PR mice (**C**). The unilateral OE was divided into four areas: dorsomedial (DM), dorsolateral (DL), ventromedial (VM), and ventrolateral (VL). Left images, low magnification; middle (HE-staining) and right images (anti-OMP staining), high magnification. Scale 300 µm at low magnification, 50 µm at high magnification. (**D**,**E**) Numbers of OSNs (**D**) and numbers of OMP-positive cells (**E**) in each area (DM, DL, VM, and VL). Significant histological changes in each area were observed between preintervention and control mice, while a significant difference between control mice and PR mice was detected only in the DM area (***P < 0.001, Mann–Whitney test).

We examined whether age-related degenerative changes in the olfactory system were similar to those seen in the auditory system. Representative coronal sections of OEs, higher magnification images of specific areas, and OMP-stained sections from 2-month-old mice on a normal diet (preintervention group), mice on a 10 month control diet (2 months of normal diet and then 10 months of control diet; control group), and PR mice (2 months of normal diet and then 10 months of PR diet; PR group) are shown in Fig. 3A–C. To analyze a broad area of the OE, coronal sections of the OE were divided into four areas (see “Materials and methods”): dorsomedial (DM), dorsolateral (DL), ventromedial (VM), and ventrolateral (VL) areas. Histological differences in the OE were not apparent between preintervention and control mice, suggesting that 10 month exposure to the custom control diet did not induce histological changes in the OE (OSNs: preintervention vs. control, DM: P = 0.13, DL: P = 0.35, VM: P = 0.65, VL: P = 0.98; OMP: preintervention vs. control, DM: P = 0.68, DL: P = 0.94, VM: P = 0.58, VL: P = 0.7; three mice per group, Mann–Whitney test, Fig. 3D,E). However, in the PR mice, the numbers of OSNs and OMP-positive cells in the DM area were significantly lower than those in the age-matched control mice, whereas significant differences were not observed in other areas (OSNs: control vs. PR, DM: P < 0.001, DL: P = 0.31, VM: P = 0.18, VL: P = 0.27; OMP: control vs. PR, DM: P < 0.001, DL: P = 0.34, VM: P = 0.11, VL: P = 0.27; three mice per group, Mann–Whitney test, Fig. 3D,E). These results indicate that PR could induce histological changes in the OE but that these effects were limited to the DM area of the OE.

**Figure 4:**
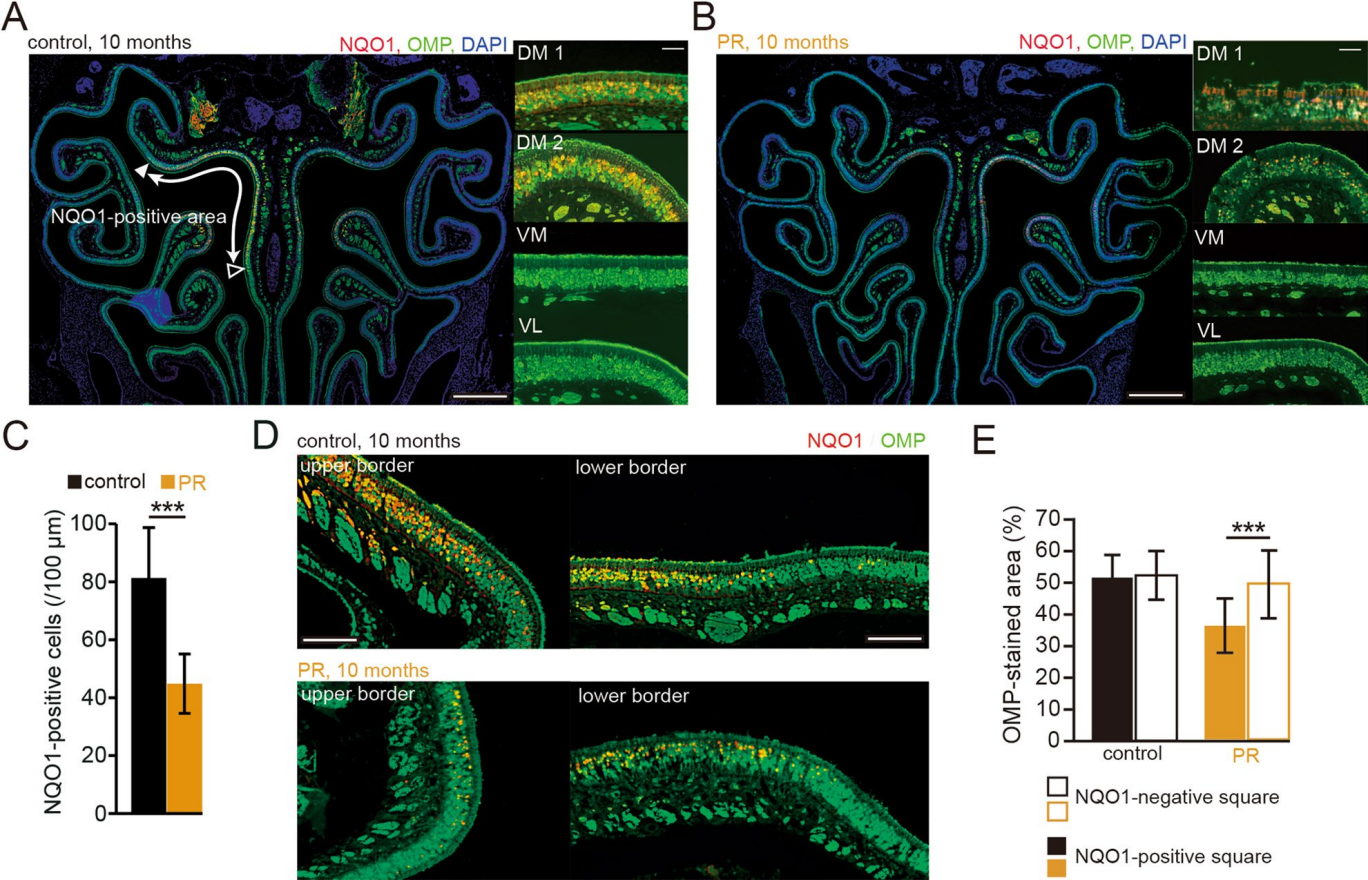
PR induces OE injury in zone 1. (**A**,**B**) Photomicrographs of representative coronal sections from control and PR mice. Low magnification images are shown on the left (red, anti-NQO1; green, anti-OMP; blue, DAPI), and high magnification images are shown on the right (red, anti-NQO1; green, anti-OMP). DM1 indicates the upper nasal septum in the dorsomedial area, and DM2 indicates the upper concha bullosa in the dorsomedial area. Closed triangle, upper border of the NQO1-positive area; open triangle, lower border of the NQO1-positive area. Scale 300 µm at low magnification, 50 µm at high magnification. (**C**) Comparison of NQO1-positive cells between control and PR mice. In PR mice, numbers of NQO1-positive cells were significantly lower than in control mice (***P < 0.001, Mann–Whitney test). (**D**) Photomicrographs of upper (closed triangle in **A**) and lower borders (open triangle in **A**) in control and PR mice (red, anti-NQO1; green, anti-OMP). Scale 50 µm. (**E**) Comparisons of the areas immunostained with OMP between the NQO1-positive and negative squares in control and PR mice. The OMP-stained area within the NQO1-positive square in the PR mice was significantly reduced compared with the NQO1-negative square (***P < 0.001, Mann–Whitney test).

The OE can be subdivided into four spatial zones (zones 1–4) according to the expression patterns of specific molecules^24,25^. The OSNs in the DM area nearly correspond to zone 1 of the OE as determined by co-localization with NQO1 expression^18^. We next examined whether the OE injury observed in the DM area could occur within zone 1 of the OE. A representative immunohistologically-stained OE from a control mouse is shown in Fig. 4A (red, NQO1; green, OMP; blue, DAPI). NQO1-positive OSNs were observed in the upper nasal septum (DM1) and upper concha bullosa (DM2), but not in other areas (Fig. 4A; VM, and VL). In the PR mice, the number of NQO1-positive cells in zone 1 (DM1 and DM2) was significantly lower than that in age-matched control mice (P < 0.001; three mice per group, Mann–Whitney test, Fig. 4B,C).

Representative high magnification images of the upper border and lower border of control and PR mice are shown in Fig. 4D. To examine detailed histological changes in the border between NQO1 positive and negative areas, we determined an NQO1-negative square (a 30 μm square) as one in which the staining was below two SDs of the mean NQO1 intensity in the NQO1 positive area, and an NQO1-positive square as just a square adjacent to the NQO1 negative square, as described in a previous report^23^. We then compared the signal intensity of the OMP-staining between the NQO1-positive and negative squares (Fig. 4E). The results showed that the OMP-staining in the NQO1-positive square was significantly less than that in the NQO1-negative square in PR mice (control: P = 0.75, PR: P < 0.001; three mice per group, Mann–Whitney test, Fig. 4E). These results indicate that 10 months of PR selectively induced OE injury in the NQO1-positive area of zone 1.

**Figure 5:**
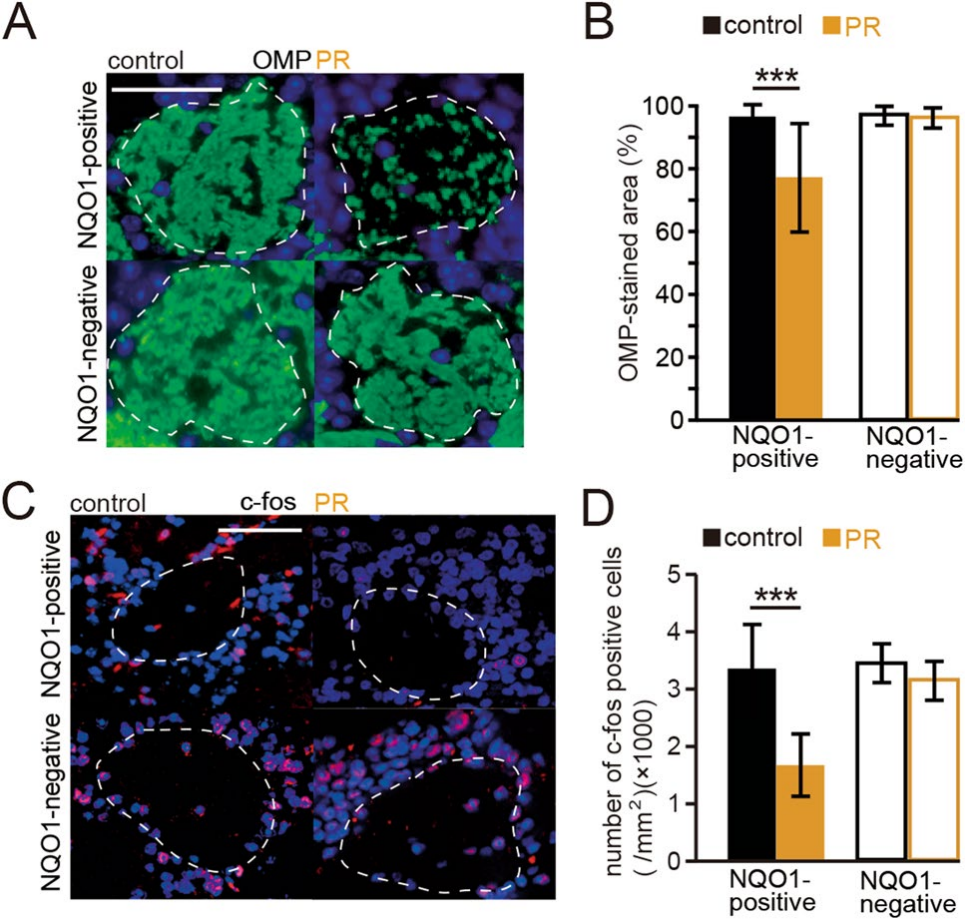
PR results in the projection of axons from fewer OSNs of zone 1. (**A**) Representative glomeruli (upper, NQO1-positive OB; lower, NQO1-negative OB) stained with anti-OMP antibody (green) from control and PR mice. Each circled area corresponds to a glomerulus. Scale 50 µm. (**B**) Summary of the ratio of areas stained with OMP. The OMP-stained area in the NQO1-positive OB of PR mice was significantly lower than that in control mice (***P < 0.001, Mann–Whitney test). However, the OMP-stained area did not differ according to condition in the NQO1-negative OB. (**C**) Representative coronal sections stained with anti-c-fos antibody in the NQO1-positive and NQO1-negative OB of control and PR mice. Aldehydes, lactones, and esters were selected as stimulus odorants to induce c-fos immunoreactivity in the OB neurons. Each circled area corresponds to a glomerulus. Scale 50 µm. (**D**) Comparison of the number of c-fos-positive cells between NQO1-positive and NQO1-negative OB in control and PR mice. The number of c-fos-positive cells (per mm^2^) in the NQO1-positive OB of PR mice was significantly less than that in control mice (***P < 0.001, Mann–Whitney test), whereas in the NQO1-negative OB of PR mice, no significant difference was detected.

**Figure 6:**
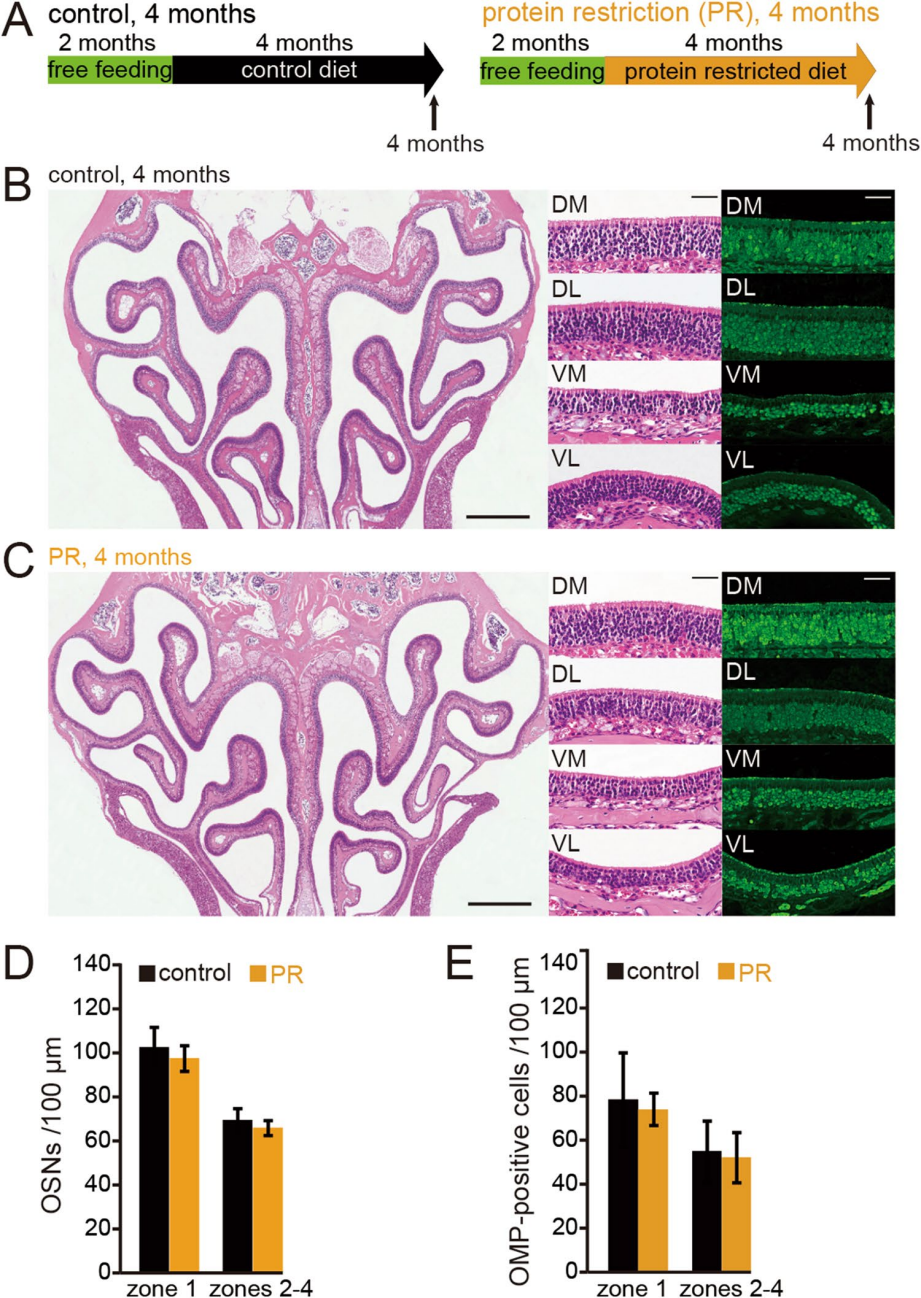
Long-term PR could be required for zone-1-specific injury. (**A**) Time course of the experimental design. Control, 4 months on control diet; PR, 4 months on protein-restricted diet. (**B**,**C**) Photomicrographs of representative coronal sections of the OE in control (**B**) and PR mice (**C**). Left images, low magnification; middle (HE-staining) and right images (anti-OMP staining), high magnification. Scale 300 µm at low magnification, 50 µm at high magnification. (**D**,**E**) Number of OSNs (**D**) and number of OMP-positive cells (**E**) in zone 1 and zones 2–4. No significant histological differences were observed between control and PR mice in any zone (Mann–Whitney test).

To examine the effect of PR on the axonal projections to the glomeruli, we measured the OMP-stained area within individual glomeruli of the OBs of control and PR mice. Representative sections of the glomerulus (upper, NQO1-positive; lower, NQO1-negative) stained with an OMP antibody are shown in Fig. 5A. In NQO1-positive OB, we observed significantly lower OMP-stained areas in PR mice than in age-matched control mice, whereas we could not detect such differences in NQO1-negative OB (NQO1-positive: PR, P < 0.001; NQO1-negative: PR, P = 0.79; three mice per group, Mann–Whitney test, Fig. 6B), suggesting that the PR intervention resulted in fewer OSNs of zone 1 projecting axons. To examine whether zone 1-specific injury of the OE functionally disrupts stable sensory inputs to the OB neurons, we examined the expression of c-fos (a neural activity marker) induced by specific odors in the NQO1-positive and negative OBs, and compared the number of c-fos-positive cells between the control and PR mice. On the basis of our previous report^26^, we selected aldehyde, lactone, and ester categories as stimulus odorants for activating a broad area of the OB. Representative sections of the OB (upper, NQO1-positive; lower, NQO1-negative) stained with an anti-c-fos antibody are shown in Fig. 5C. In NQO1-positive OB, we observed a significantly lower number of c-fos-positive cells in PR mice than in age-matched control mice, whereas in NQO1-negative OB, we could not detect significant differences in c-fos-positive cells between age-matched control and PR mice (NQO1-positive: P < 0.001; NQO1-negative: P = 0.13; four mice per group, Mann–Whitney test; Fig. 5D). These results suggest that the decreased number of OMP-positive cells and their axonal projections to the glomeruli in OSNs of zone 1 parallels the decrease in the glomerular responses to odorants, implying functional disruption of stable inputs to the OB.

### A long-term period of PR is required for zone 1-specific injury

To examine whether short-term PR could induce zone 1-specific injury in the OE, we observed the histological changes of the OE following 4 months of PR (Fig. 6A). A representative coronal section of the whole OE, and higher magnification images of specific areas and OMP-stained sections following 4 months of control and PR diet, are shown in Fig. 6B,C, respectively. For each of the zones 1–4 within the OE, no significant differences in the numbers of OSNs and OMP-positive cells were found between PR mice and age-matched control mice (OSNs [zone 1]: P = 0.1; [zones 2–4]: P = 0.17; OMP [zone 1]: P = 0.73; [zones 2–4]: P = 0.55; three mice per group, Mann–Whitney test, Fig. 6D,E). Although we cannot rule out the possibility that 4 months of PR caused metabolic alterations because the body weight gain significantly decreased following 5 months of PR (Fig. 1), these results indicate that a long period (> 4 months) of PR is required to induce apparent histological changes in zone 1 of the OE.

### PR induces up-regulation of proliferation in progenitor basal cells but more apoptotic OSNs

**Figure 7:**
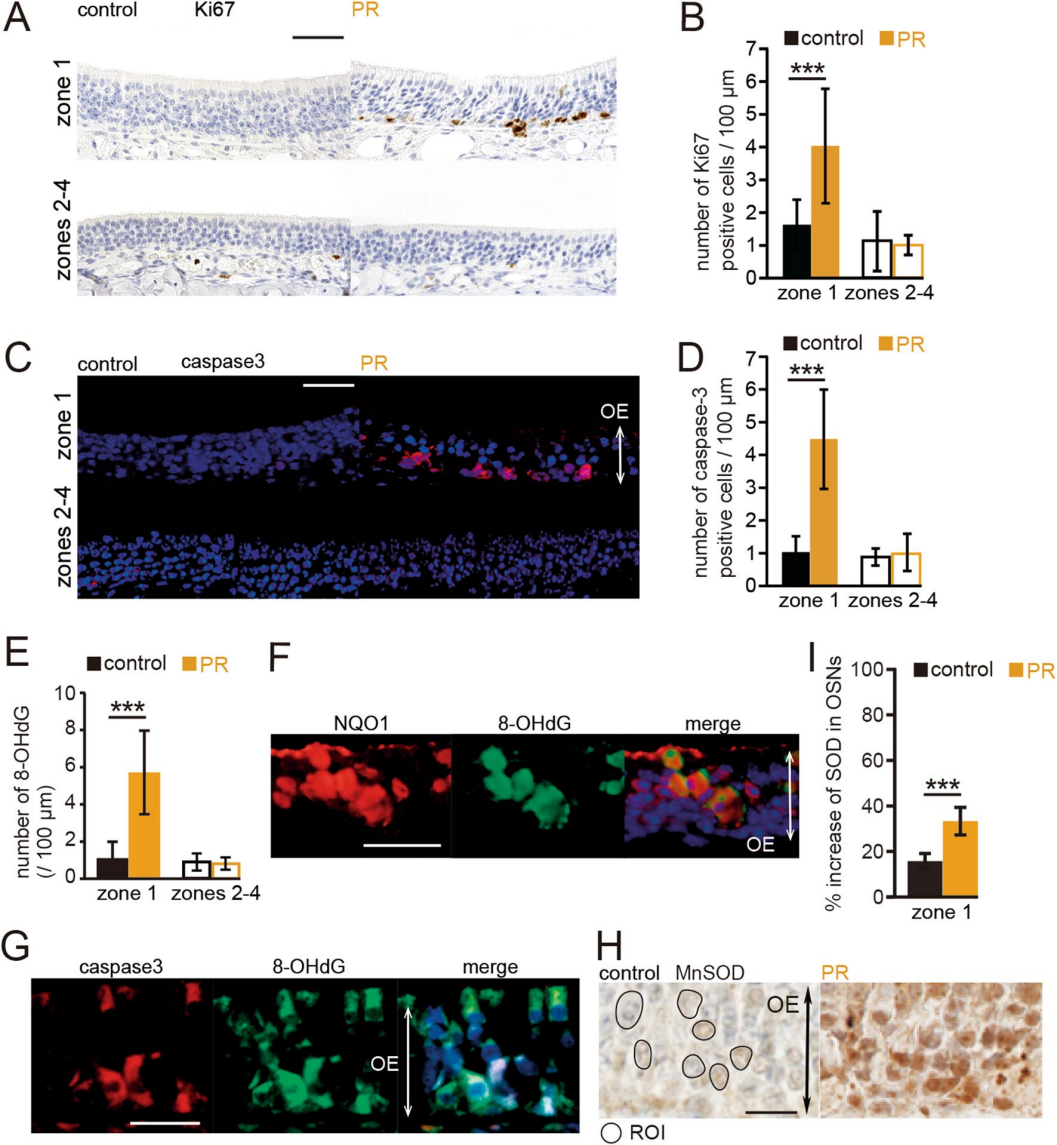
8-OHdG production occurred substantially in NQO1-positive cells and could be involved in the apoptosis of OSNs in zone 1. (**A**) Representative images of Ki67-positive cells of zone 1 and zones 2–4 in control and PR mice. Scale bar 50 μm. (**B**) The number of Ki67-positive cells in zone 1 and zones 2–4 in control and PR mice. In zone 1, the number of Ki-67-positive cells in PR mice was significantly higher than that in control mice (***P < 0.001, Mann–Whitney test), whereas in zones 2–4, the number of Ki67-positive cells in PR mice was not significantly different from that in control mice. (**C**) Representative images of the c-fos-positive cells of zone 1 and zones 2–4 in control and PR mice. *OE* olfactory epithelium. Scale bar 50 μm. (**D**) The number of c-fos-positive cells in zone 1 and zones 2–4 in control and PR mice. In zone 1, the number of c-fos-positive cells in PR mice was significantly higher than that in control mice (***P < 0.001, Mann–Whitney test), whereas in zones 2–4, the number of c-fos-positive cells in PR mice was not significantly different to that in control mice. (**E**) The number of 8-OHdG-positive cells in zone 1 and zones 2–4 in control and PR mice. The number of 8-OHdG-positive cells in PR mice was significantly higher than that in control mice (***P < 0.001, Mann–Whitney test). (**F**) Representative images stained with anti-NQO1 antibody (red) and anti-8-OHdG antibody (green) in PR mice. Most of the 8-OHdG-positive cells were co-stained with anti-NQO1 antibody. *OE* olfactory epithelium. Scale bar 30 µm. (**G**) Representative images stained with anti-caspase 3 antibody (caspase3, red) and anti-8-OHdG antibody (green) in PR mice. A majority of the 8-OHdG-positive cells were co-stained with anti-caspase 3 antibody. Scale bar 30 µm. (**H**) Representative images stained with anti-MnSOD antibody in control and PR mice. In this analysis, we measured the intensity of the immunostaining in the OSNs (circle) for anti-MnSOD antibody and calculated the relative intensity of the immunostaining in the OSNs compared with axon bundles. OE, olfactory epithelium. ROI, region of interest. Scale bar, 20 µm. (**I**) Summary of the percent increases of MnSOD-immunostaining in OSNs in control and PR mice. The relative intensities of OSNs in PR mice were significantly higher than those in control mice (***P < 0.001, Mann–Whitney test).

The lower numbers of OSNs in zone 1 could be explained by lower OSN proliferation, higher cell death, or both. To evaluate these possibilities, we examined Ki67-positive cells (a cell marker of proliferation) and caspase-3-positive cells (a cell marker of cell death) in the OE of control and PR mice. Figure 7A shows representative images stained with anti-Ki67 antibody in control and PR mice (upper, zone 1; lower, zones 2–4). The number of Ki67-positive cells in zone 1 was significantly higher in the PR mice than in the age-matched control mice, while this difference was rarely observed in zones 2–4 (zone 1: P < 0.001; zones 2–4: P = 0.68; four mice per group, Mann–Whitney test, Fig. 7B). These results argue against the idea of down-regulation of proliferation in zone 1 progenitor basal cells. Figure 7C shows representative images stained with anti-caspase3 antibody from control and PR mice (upper, zone 1; lower, zones 2–4). As expected, zone 1 showed prominently higher numbers of caspase-3-positive cells in PR mice than in age-matched control mice, whereas this difference was rarely observed in zones 2–4 (zone1: P < 0.001; zones 2–4: P = 0.9; four mice per group, Mann–Whitney test, Fig. 7D). These results indicate that PR induced a prominent increase in apoptotic OSNs with a basal cell proliferative response in zone 1, resulting in incomplete replacement of OSNs within this zone.

### Oxidative stress in OSNs of zone 1 is strongly linked to NQO1 activity

NQO1 is a cytosolic flavoenzyme and generally acts to protect against oxidative stress induced by a variety of metabolic situations, including metabolism of quinones and other xenobiotics ^27^. However, NQO1 can occasionally mediate ROS generation by conjugation with superoxide dismutase (SOD), leading to enhanced oxidative stress^20^. We therefore hypothesized that increased ROS generation mediated by NQO1 activity induced oxidative stress, resulting in an increase in apoptotic cells in zone 1 of the OE. We therefore examined 8-hydroxydeoxyguanosine (8-OHdG) expression in zone 1 of the PR mice as a marker of oxidative stress. PR induced prominently increased numbers of 8-OHdG-positive cells in zone 1 (8-OHdG: P < 0.001; three mice per group, Mann–Whitney test, Fig. 7E). To examine whether increased oxidative stress could be involved in the NQO1 activity, we co-labeled OSNs with anti-NQO1 and anti-8-OHdG antibodies. Most of the 8-OHdG-positive cells were also co-labeled with NQO1 (167 of 179, 93.3%, three mice, Fig. 7F). These results indicate that increased oxidative stress occurs substantially within the NQO1-positive cells. To examine whether increased oxidative stress within zone 1 could induce apoptosis of OSNs, we co-labeled OSNs with anti-caspase-3 and anti-8-OHdG antibodies. We found that a majority of the 8-OHdG-positive cells were co-labeled with caspase-3 (256 of 284, 90.1%, three mice, Fig. 7G). Although we cannot directly demonstrate that oxidative stress induced cell death in zone 1, the high occurrence of co-staining results indicates that increased oxidative stress could be involved in the apoptosis of OSNs in zone 1 in the PR mice. We further examined whether SOD activity of zone 1 could be enhanced, in support of our hypothesis that ROS generation mediated by NQO1 activity could be involved in the enhanced SOD. SODs are present as three isoforms in mammals: cytoplasmic Cu/Zn-SOD, mitochondrial MnSOD, and extracellular Cu/ZnSOD^28^. Because mitochondria are a main source of ROS, we focused on MnSOD in this analysis. To quantitively evaluate MnSOD activity in the OE, we measured the intensity of immunostaining for anti-MnSOD antibody. We defined the intensities within axon bundles as control areas, and then compared the relative intensity of immunostaining between the OSNs and the axon bundles in zones 2–4, because the OE structure in zone 1 is prone to collapse because of injury. The results showed that the relative intensities of each condition were significantly higher than those in the age-matched control mice (P < 0.001; three mice per group, Mann–Whitney test, Fig. 7H,I). These results indicate that enhanced oxidative stress in PR mice is strongly involved in the bioactivation of NQO1 under enhanced MnSOD activity, leading to prominent cell death of OSNs in zone 1.

## Discussion

In the current study, we investigated the histological and functional effects of long-term PR on cell dynamics in the OE. We found that PR intervention induced a significant decrease in OSNs in the DM area of the OE, which corresponds to zone 1, as determined by co-localization with NQO1. Increased proliferative basal cells together with more apoptotic OSNs were observed in zone 1. Furthermore, oxidative stress was significantly increased in NQO1-positive cells and apoptotic OSNs. These results indicate that PR could induce structural and functional damage targeted to zone 1 of the OE.

With regard to sex preference and mouse strains, we used C57BL6 male mice in the present study because of the following reasons. First, female rodents show more variable research results than males because of hormonal fluctuations between different phases of the reproductive cycle^29^. Hormonal fluctuations associated with the female’s reproductive cycle can affect insulin signaling, nutrient metabolism in response to nutritional imbalance^30^, modulation of gene expression in the brain^31^, and auditory neural responses^32,33^. Accordingly, it is possible that female rats or mice could show more variability and less reproducibility than males in neuroscience experiments. Second, the C57BL/6J mouse strain is a long-lived strain (mean lifespan of approximately 30 months) and is the most widely used mouse model for studying aging and age-associated diseases^34,35^. Third, the C57BL/6 mouse strains carry a specific mutation (Cdh23^753A^) in the Cdh23 gene that encodes a component of the hair-cell tip link and is known to promote the early onset of AHL^36,37^. Furthermore, hair cells in mice carrying the Cdh23^753A^ mutation are more susceptible to oxidative stress and apoptosis^35^. By contrast, the CBA/J mouse strain, which does not possess the Cdh23^753A^ mutation, displays late-onset AHL by 18 months of age^38,39^. Given these characteristics, we think that the C57BL/6J male mouse strain is an appropriate choice for studying the effects of PR on AHL over a duration of 10 months.

In the auditory systems of mice and humans, age-related hearing loss (AHL) generally affects the basal turn of the cochlea more profoundly than it effects the apical and middle turns^21,40^. It is reported that such age-related degenerative changes in the cochlea are reduced by some non-genetic interventions other than PR. For example, long-term voluntary exercise significantly delayed the progression of AHL and reduced OHC loss in the apical turn of the cochlear in aged mice^23,41^. Long-term CR of 10 month duration suppressed apoptotic cell death in the apical turn of the cochlea compared with non-CR mice^23,42^. In an analogy with these interventions, PR also had preventive effects on age-related degenerative changes in the OHCs in the apical turn. In striking contrast to the positive effects of PR on the auditory system, negative functional and structural effects were observed on the olfactory system.

The key switches that respond to long-term PR include mTORC1 (mechanistic target of rapamycin complex 1), autophagy, GH (growth hormone)/insulin/IGF1 (insulin-like growth factor 1), and FGF21 (fibroblast growth factor 21) signaling pathways^43,44^. These pathways respond to decreased levels of amino acids and are expected to act to reduce anabolic responses and oxidative stress^11,12^. However, immunoreactivity of the oxidative stress marker 8-OHdG was significantly greater in zone 1 than in zones 2–4, suggesting that there might be different cellular mechanisms regulating the generation of oxidative stress between zone 1 and zones 2–4. The generation of higher oxidative stress in zone 1 could be associated with NQO1 activity in biochemical processes conjugated with SOD because of the co-localization of NQO1 expression and cell damage.

NQO1 is a cytosolic flavoenzyme and catalyzes two-electron reduction of quinones and aromatic nitrocompounds. NQO1 can generally protect cells against the toxicity of some quinones. However, bioactivation of NQO1 within metabolic pathways involving multiple enzymes is not a simple process, and NQO1 with conjugation of SOD facilitates ROS generation through the reaction of some unstable hydroquinones with oxygen, leading to the generation of oxidative stress^20,45,46^. We speculate that the physiological properties of NQO1 under enhanced SOD activity may induce high susceptibility of OSNs to injury from environmental quinone agents or endogenously produced neurotoxins. Although SOD could work as an antioxidant enzyme, imbalance between ROS generation and antioxidant defenses within zone 1 might induce continuous oxidative damage, which could in turn result in structural and functional injury of zone 1 OSNs, regardless of the regeneration of new neurons. In addition, some pathological conditions, such as intraperitoneal injections of olfactory toxins and inhalation of hydrogen sulfide, induce neural degeneration of the OSNs predominantly localized in zone 1^15^. These observations imply that NQO1 may also increase susceptibility to the harmful action of other types of substrates. Further study will be needed to clarify the detailed physiological properties of NQO1 under the aging process and its role in various pathological conditions.

The PR mice in the current study were subjected to a 30% restriction in the casein proportion of their manufactured diet. Casein is a high-quality protein and contains a high proportion of methionine*.* Several studies have reported positive effects of methionine restriction on metabolic health^11,47,48^. Methionine promotes metabolic hormone secretion, while diets with lower levels of methionine decrease circulating insulin and IGF-1, thereby promoting long-term health. Accordingly, other possible mechanisms of zone-specific injury may be associated with the reduction of insulin/IGF1 signaling triggered by decreased methionine at the systemic level.

OSNs express high levels of mRNA for metabolic hormone receptors^49–52^. When targeting their receptors, metabolic hormones substantially affect neuron survival and activity^53–56^. For example, insulin/IGF1 increases the number of cultured OSNs in vitro, and in adult rats after injury, it has an anti-apoptotic effect on OSNs through activation of the intracellular cAMP signaling cascade^57,58^. Thus, PR could affect the regulation of cell dynamics in adult OE under decreased levels of insulin/IGF1 signaling, and such decreased signaling might facilitate high susceptibility of zone 1 OSNs to injury.

New treatment strategies aimed at preventing oxidative stress-related OSN injury might be required in the future. A previous report indicated that *N*-acetylcysteine has antioxidant effects, causing alterations to the expression of genes involved in the oxidative stress pathway, and *N*-acetylcysteine application significantly inhibited OE degeneration after bulbectomy and methimazole-induced injury in in vivo and in vitro experiments^59^. Insulin is another candidate for a possible drug for preventing OE injury. Insulin receptors are expressed in the OE, and their activation is involved in the phosphoinositide 3-kinase–AKT–forkhead box protein O and RAS-MAPK pathways, which ultimately affect key cellular processes such as protein synthesis, autophagy, apoptosis, and resistance to oxidative stress^60^. Accordingly, the application of these drugs into the nostril might be an effective therapeutic strategy to prevent OE injury caused by PR.

**Figure 8:**
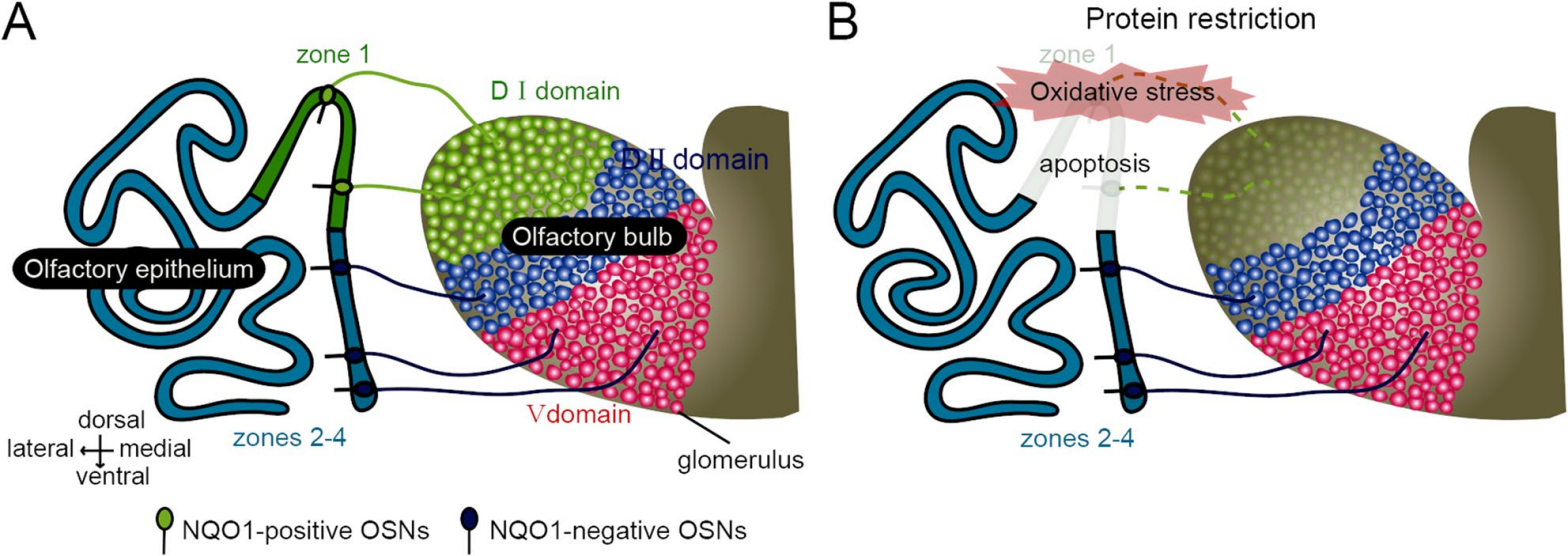
Schematic diagrams of olfactory information flow from OSNs to OB domains. (**A**) Mouse olfactory receptors (ORs) are divided into class I and class II receptors based on phylogenetic analysis. Class I OSNs expressing class I ORs are substantially distributed within zone 1 and mediate odor information to the dorsal domain of the OB (D_I_ domain), whereas class II OSNs expressing class II ORs are distributed within zones 2–4, and mediate odor information to the dorsal domain of the OB (D_II_ domain) and ventral domain of the OB (V domain). (**B**) Olfactory information flow from OSNs in zone 1 and zones 2–4 to OB domains under PR intervention. Under the PR intervention, oxidative stress occurred substantially in the NQO1-positive cells and induced zone 1-specific injury as apoptosis. Class I OSNs projecting to the D_I_ domain are responsible for innate aversive behavior to odorants produced from spoiled foods. As a result, neural circuits mediating the induction of innate aversive behavior could be functionally damaged.

Mouse odorant receptors (ORs) are divided into class I and class II receptors according to phylogenetic analysis^61^. Class I OSNs expressing class I ORs are substantially distributed within zone 1, and their axons converge onto the dorsomedial region of the OB (a dorsal domain for class I odorant receptors, D_I_ domain). By contrast, class II OSNs expressing class II ORs are broadly distributed within zones 2–4, and their axons converge onto the dorsolateral (a dorsal domain for class II odorant receptors, D_II_ domain) and ventral regions of the OB (a ventral domain for class II odorant receptors, V domain; Fig. 8A)^62^. Furthermore, it has been reported that the class-specific anatomical domain organization in the OB correlates with functional odor-induced innate responses. Class I OSNs projecting to the D_I_ domain are responsible for innate aversive behavior to odorants produced from spoiled foods, while class II OSNs projecting to the D_II_ domain are responsible for innate fear responses to predator odors^62,63^. Thus, selective reduction of OSNs in zone 1 under PR intervention is expected to decrease sensitivity to spoiled foods and thus increase the odor threshold for the induction of aversive behavior (Fig. 8B). In wild animals, high sensitivity and subsequent appropriate reactions to danger-signaling odors are critical behavior for survival and adaptation to the external environment. This critical trade-off between the prevention of age-related degenerative changes in the auditory system and structural and functional damage targeted to zone 1 in the olfactory system may adversely affect species perpetuation. Determination of the appropriate amount of PR will be required to maximize long-term metabolic health and preventive effects against age-related degeneration of the cochlear while minimizing injury of the OE.

## Materials and methods

### Animals

Male C57BL/6 (8-week-old, N = 16) mice were used for the experiments and were kept in a 12:12 h light/dark cycle at 21–22 °C. The mice were maintained on a standard purified mouse diet for 2 months prior to the start of the experiment. The 2-month-old mice were randomly allocated to non-restricted control (control) and protein-restricted (PR) groups, which were then given two different dietary compounds (Table 1).

### Protein restriction

Two custom diets based on the AIN-93M (American Institute of Nutrition) formulation were manufactured by a local stock-feed company^64^ (Oriental Yeast Co., Tokyo, Japan). Both diets, which varied in protein composition only (Table 1), were presented as dry pellets every 2 days in a quantity determined according to the daily food intake data (approximately 4 g per day). The PR mice were fed with a diet of 353.8 kcal/100 g, in which the percentage of casein was 30% lower than the AIN-93M level, with it being replaced by cornstarch. The control mice were fed with a diet of 353.6 kcal/100 g.

### Analysis of hair cell and spiral ganglion cell numbers

Hair cells (HCs) were counted as present if the cell body and cuticular plate remained intact. The numbers of remaining HCs at the basal and apical turns were counted for at least 10 sections per animal^65^. The inner hair cell (IHC) and outer hair cell (OHC) survival rates were calculated using the following formulae: IHC survival rate % = 100 × (the number of present IHCs in examined specimens/the number of examined specimens); OHC survival rate % = 100 × (the number of present OHCs in examined specimens/the number of examined specimens)^23,65^. The spiral ganglion cell (SGC) densities (number/mm^2^) at each turn of each animal were calculated by dividing the number of SGCs by the area of Rosenthal’s canal measured using ImageJ software (NIH)^65^.

### Immunohistochemistry

Following methods described in our previous reports^23,26^, mice were perfused with 4% paraformaldehyde in a 0.1% phosphate buffer, sacrificed, and post-fixed for 24 h in the same fixative. The head tissues, including the cochlear, OE, and olfactory bulb (OB), were decalcified with 10% EDTA solution (pH 7.0) and embedded in paraffin. Four-μm thick coronal sections were cut and mounted on silane-coated slides. Deparaffinized sections were autoclaved at 121 °C for 20 min in Target Retrieval Solution (S1700; Dako) for antigen retrieval.

Immunohistochemistry was performed using one or two of the following antibodies: anti-olfactory marker protein (OMP, goat polyclonal, 1:2000 dilution; Wako Chemicals; Cat#544-10001-WAKO; RRID: AB_664696), anti-NQO1 (rabbit polyclonal, 1:300; Cell Signaling Technology; Cat# HPA007308; RRID: AB_1079501), anti-activated caspase-3 (rabbit polyclonal, 1:300; Cell Signaling Technology; Cat#9661; RRID: AB_2341188), anti-Ki-67 (rabbit monoclonal, 1:300; Lab Vision; Cat#RM-9106-S1; RRID: AB_149792), anti-c-fos antibody (rabbit polyclonal, 1:50; Santa Cruz Biotechnology; Cat#2250; RRID: AB_2247211), anti-MnSOD antibody (rabbit monoclonal, 1 : 100 dilution; Epitomics, Inc.; Cat#ab13533; RRID: AB_300434), and anti-8-hydroxy-2′-deoxyguanosine antibody (8-OHdG, goat polyclonal antibody, 1 : 100 dilution; Alpha Diagnostic International, Inc.; Cat#ABIN285804, RRID: AB_10778260).

The immunoreaction was detected using one or two of the following antibodies from the Histofine Simple StainMAX-PO secondary antibody systems (Nichirei), according to the manufacturer’s instructions: donkey anti-goat Alexa Fluor 488 (1:100; Invitrogen) and donkey anti-rabbit Alexa Fluor 594 (1:100; Invitrogen), applied for 1 h at room temperature^23^.

### Analysis

For each OE, three coronal sections located between the caudal and rostral regions were examined, and each section was cut at 500-μm intervals. The numbers of OSNs labeled by anti-OMP, anti-NQO1, anti-caspase3, and anti 8-OHdG antibodies, and the number of basal cells labeled by anti-Ki67 antibody, were quantitatively analyzed using sections with single or double immunostaining for each antigen, and counterstaining with DAPI or hematoxylin. Immunostaining-positive cells were considered to be those showing significant staining that exceeded two standard deviations (SDs) of the mean background intensity of the connective tissue under the lamina propria^23,26^. Coronal sections of the OE were divided into medial and lateral areas between the most lateral regions of the OE and the nasal septum. The lateral and medial areas were further divided into upper and lower regions between the most dorsal and ventral edges of the OE, providing four areas in each coronal section of the OE: dorsolateral (DL), dorsomedial (DM), ventrolateral (VL), and ventromedial (VM) areas^23^. The numbers of OSNs and immunostained cells (OMP-, NQO1-, Ki67-, caspase-3-, and 8-OHdG-positive cells) in 300 μm of each area or each zone (zone 1, zones 2–4) on both right and left sides were counted. The mean ± SD per 100 μm OE length was then calculated for the numbers of OSNs, OMP-, Ki67-, caspase-3-, and 8-OHdG-positive cells in each group^26^.

For analysis of the border between NQO1 positive and negative areas (Fig. 4D,E), an NQO1-negative square was defined as one in which the staining was below two SDs of the mean NQO1 intensity in the area clearly identified as NQO1-positive OE. An NQO1-positive square was defined as a 30 μm square adjacent to an NQO1-negative square. The signal intensities of the OMP-staining were compared between NQO1-positive and negative squares^23^.

For each OB, three coronal sections were selected, and then at least five glomeruli from NQO1-positive and negative areas in the OB section were randomly selected (Fig. 5). A significant OMP-stained area was defined as one in which the staining exceeded two SDs of the mean background intensity in the external plexiform layer of the OB^23^. The percentage of significantly OMP-stained areas within a glomerulus was calculated by dividing the area with significant OMP-staining by the total glomerulus area (OMP-stained area/glomerulus area × 100)^23,26^. Analysis of the immunostained areas was performed using ImageJ software (NIH).

To analyze the co-staining with anti-8-OHdG and anti-NQO1 antibody, or anti-8-OHdG and anti-caspase-3 antibody (Fig. 7), double-positive cells in zone 1 were counted under a fluorescence microscope at a magnification of 40× for each group.

The intensity of the immunostaining for anti-MnSOD antibody was measured to quantitatively evaluate the MnSOD activity in the OE^23^. Seven OSNs (three mice per group, circle, Fig. 7H) and seven axon bundles as control areas were randomly selected, and the averaged signal intensities of these OSNs and axon bundles were calculated. The relative signal intensity of the MnSOD-stained areas was calculated by dividing the average signal intensity in the OSNs by the average signal intensity in axon bundles (signal intensity of MnSOD in OSNs/signal intensity of MnSOD in axon bundles × 100)^23^. The signal intensity analysis was performed using ImageJ software (NIH). The analyses were made by observers who were blind to the identity of each specimen.

### Odor-induced c-fos expression in the OB

Following methods described in our previous report^26^, mice were housed individually in isolation boxes and supplied with pure air that was deodorized through a charcoal filter. The mice were kept in new cages without food pellets for 4 h before odor application (three mice per group). Odorants in the three categories of aldehydes (propyl aldehyde, n-valeraldehyde, n-heptylaldehyde, benzaldehyde, and perilla aldehyde), lactones (γ-butyrolactone, γ-heptalactone, δ-hexalactone, δ-nonalactone, and Y-octalactone), and esters (amyl hexanoate, b-γ-hexenyl acetate, terpinyl acetate, and isoamyl acetate) were diluted to a 1/10 concentration with mineral oil, and a cotton sheet soaked with 100 μl of diluted solution was placed in a dish. The odor was applied by placing the dish in the cage twice for 1 h each time, with a 10-min interval between placements. After the last odor application, the mice were perfused with fixative and subjected to an analysis of c-fos expression in the OB. As the primary antibodies were derived from the same animal species, we selected two adjacent slices (4-μm interval) and immunostained each one with different antibodies. One slice was immunostained with anti-NQO1 antibody for definition of the DI domain (NQO1-positive) and DII/V domains (NQO1-negative) in the OB, whereas the other slice was immunostained with anti-c-fos antibody, and c-fos-positive cells were counted by referencing the domain structure. We focused on c-fos expression within the glomerular layer because the increase in c-fos expression within the glomerular layer is region-specific. In each domain, c-fos-positive cells were counted under a fluorescence microscope at a magnification of 40×, and the results were expressed as the number of c-fos-positive cells per mm^2^.

### Statistical analysis

Statistical analyses of the differences between control vs. PR mice or control vs. preintervention mice were performed with the Mann–Whitney *U-*test because normality in each group could not be ensured (Figs. 1C, 2C–D,F, 3D–E, 4C,E, 5B,D, 6D–E, 7B,D–E,I). Error bars indicating mean ± standard deviation (SD) are provided. P values < 0.05 were considered statistically significant.

### Study approval

All animal studies were approved by the Experimental Animal Research Committee at the University of Tokyo, and the animal study methods were carried out in accordance with the approved guidelines.
